# Optimizing cisplatin delivery to triple-negative breast cancer through novel EGFR aptamer-conjugated polymeric nanovectors

**DOI:** 10.1186/s13046-021-02039-w

**Published:** 2021-07-22

**Authors:** Lisa Agnello, Silvia Tortorella, Annachiara d’Argenio, Clarissa Carbone, Simona Camorani, Erica Locatelli, Luigi Auletta, Domenico Sorrentino, Monica Fedele, Antonella Zannetti, Mauro Comes Franchini, Laura Cerchia

**Affiliations:** 1 Institute of Experimental Endocrinology and Oncology “Gaetano Salvatore”, CNR, Via S. Pansini 5, 80131 Naples, Italy; 2 University of Campania “L.Vanvitelli” Department of Precision Medicine, S. Andrea delle Dame - Via L. De Crecchio, 7 - 80138 Naples, Italy; 3 Department of Industrial Chemistry Toso Montanari, University of Bologna, Viale Risorgimento 4, 40136 Bologna, Italy; 4 Institute of Biostructure and Bioimaging, CNR, Via T. De Amicis 95, 80145 Naples, Italy; 5 Ceinge-Biotecnologie Avanzate s.c.a.r.l, via Gaetano Salvatore 486, 80145 Naples, Italy

**Keywords:** Aptamer, Cancer targeting, EGFR, Enhanced therapeutic effects, Nanomedicine, Targeted drug delivery, TNBC

## Abstract

**Background:**

Management of triple-negative breast cancer (TNBC) is still challenging because of its aggressive clinical behavior and limited targeted treatment options. Cisplatin represents a promising chemotherapeutic compound in neoadjuvant approaches and in the metastatic setting, but its use is limited by scarce bioavailability, severe systemic side effects and drug resistance. Novel site-directed aptamer-based nanotherapeutics have the potential to overcome obstacles of chemotherapy. In this study we investigated the tumor targeting and the anti-tumorigenic effectiveness of novel cisplatin-loaded and aptamer-decorated nanosystems in TNBC.

**Methods:**

Nanotechnological procedures were applied to entrap cisplatin at high efficacy into polymeric nanoparticles (PNPs) that were conjugated on their surface with the epidermal growth factor receptor (EGFR) selective and cell-internalizing CL4 aptamer to improve targeted therapy. Internalization into TNBC MDA-MB-231 and BT-549 cells of aptamer-decorated PNPs, loaded with BODIPY505-515, was monitored by confocal microscopy using EGFR-depleted cells as negative control. Tumor targeting and biodistribution was evaluated by fluorescence reflectance imaging upon intravenously injection of Cyanine7-labeled nanovectors in nude mice bearing subcutaneous MDA-MB-231 tumors. Cytotoxicity of cisplatin-loaded PNPs toward TNBC cells was evaluated by MTT assay and the antitumor effect was assessed by tumor growth experiments *in vivo* and *ex vivo* analyses.

**Results:**

We demonstrate specific, high and rapid uptake into EGFR-positive TNBC cells of CL4-conjugated fluorescent PNPs which, when loaded with cisplatin, resulted considerably more cytotoxic than the free drug and nanovectors either unconjugated or conjugated with a scrambled aptamer. Importantly, animal studies showed that the CL4-equipped PNPs achieve significantly higher tumor targeting efficiency and enhanced therapeutic effects, without any signs of systemic toxicity, compared with free cisplatin and untargeted PNPs.

**Conclusions:**

Our study proposes novel and safe drug-loaded targeted nanosystems for EGFR-positive TNBC with excellent potential for the application in cancer diagnosis and therapy.

**Supplementary Information:**

The online version contains supplementary material available at 10.1186/s13046-021-02039-w.

## Background

Breast cancer (BC) is the most commonly diagnosed type of cancer in women and is the main cause of cancer-related deaths. Based on the expression of estrogen receptor (ER), progesterone receptor (PR) and epidermal growth factor receptor 2 (HER2/ErbB2), BC can be classified into three main groups: hormone receptor positive, HER2-enriched and triple-negative (TNBC) [1]. TNBC, which represents approximately 15–20 % of all diagnosed BCs, more frequently affects younger patients, usually appears in the form of high-grade invasive ductal carcinoma and is associated with poorer prognosis compared to other BC subtypes [2, 3]. TNBC is highly invasive and many patients will have distant metastases that often occur in the third year after diagnosis and generally involve the brain and visceral organs [4]. Without the possibility to use therapies against ER, PR and ErbB2, and only two recently approved targeted therapies available for a limited group of patients [5, 6], conventional chemotherapy represents the solely systemic treatment used for most TNBC patients in both first-line settings and advanced stages of the disease [7–9]. Nevertheless, chemotherapy efficacy is considerably limited by the low stability, poor bioavailability and high-dose requirements of the nonselective drugs, leading to toxicity to normal tissues and occurrence of multiple drug resistance [10–12]. Therefore, developing chemotherapeutics that specifically target cancer cells, thus reducing adverse side effects while improving therapeutic efficacy, is highly desirable. Targeted biocompatible nanovectors presenting multiple therapeutic functions have great potential for the treatment of cancer and nanomedicine (the application of nanotechnology to medicine) holds great promise for cancer treatment due to the possibility of tailoring the synthesis of nanoparticles in order to produce particles with narrow size distributions and cavities where drugs can be incorporated. Active targeting may be achieved by conjugating chemotherapeutics-containing nanovectors to high-selective recognition molecules, such as antibodies, peptides or oligonucleotide aptamers, against antigens overexpressed on cancer cells or components of the tumor microenvironment [13, 14]. This assures the precise delivery of cytotoxic payloads to cancer cells and improves both stability and activity of traditional drugs. Thanks to their safety and lack of toxicity, a wide range of nanoparticles based on Food and Drug Administration (FDA)-approved biodegradable polymers, such as poly lactic-co-glycolic acid (PLGA), is applied as nanomedicine for optimizing the delivery of cytotoxic agents to cancer [15–17].

Despite no single best target has been identified for TNBC, the epidermal growth factor receptor (EGFR) is overexpressed in ~ 60 % of TNBCs and successfully used as a dock for drug delivery approaches to these tumors, which unfortunately are resistant to tyrosine kinase inhibitors or monoclonal antibodies against the receptor [18]. We previously developed through cell-SELEX a nuclease resistant 2’Fluoro-pyrimidines (2’F-Py) RNA aptamer, named CL4, capable of binding strongly and specifically to the extracellular domain of human EGFR expressed on cancer cells of glioblastoma (GBM) [19, 20], non-small-cell lung cancer (NSCLC) [21], HER2-positive BC [22, 23] and TNBC [24]. According to the exquisite selectivity of CL4 for EGFR-positive cancers [25], the ability to actively internalize into target cells *via* receptor mediated endocytosis [19] and the nearly undetectable toxicity and immune responses in mice [20], this aptamer has been recently used to functionalize nanoparticles for targeted delivery of therapeutic oligonucleotides [26–28] or conventional paclitaxel [29] to TNBC.

In this work, we propose novel EGFR aptamer-conjugated PLGA-based nanovectors as a system to actively deliver cisplatin (Cis-Pt), a high efficacious anticancer drug whose clinical application is however limited by poor bioavailability and high toxicity, to TNBC. Confocal microscopy and *in vivo* imaging analyses of TNBC xenografts demonstrated the targeting ability of CL4-equipped nanoparticles to EGFR-positive tumor cells. Cis-Pt was efficiently encapsulated in the targeted nanovectors that caused significant higher cytotoxicity than naked drug and nanovectors unconjugated or conjugated with a scrambled aptamer. Also, our CL4-based nanovectors achieved higher *in vivo* antitumor efficacy than free drug and untargeted PNPs without any signs of systemic toxicity. Thus, these results suggest a new nanoplatform delivering an excellent chemotherapeutic agent to cancer cells tagged with one of the most widely overexpressed oncoprotein on human epithelial cancers [30].

## Methods

### Aptamers

The sequences of the 2’F-Py RNA EGFR CL4 and scrambled (SCR) aptamer, used as negative control, were previously reported [19]. NH_2_-terminated aptamers were synthesized by LGC Biosearch Technologies (Risskov-Denmark).

### Synthesis of nanoparticles

PLGA-block-polyethylene glycol (PLGA-b-PEG) nanoparticles where prepared following a previously reported protocol [31] with slight modifications. Briefly, 100 mg of PLGA-b-PEG-COOH, synthesis already reported [32], were dissolved in 36 ml of chloroform and admixed to 3.6 ml of dimethylformamide and H2O (ratio 1:1.4) containing the hydrophilic drug Cis-Pt (Sigma-Aldrich, Milan, Italy). Then the two-phase mixture was emulsified, in an ice bath, with a tip probe sonicator (600 W input, 50 % ampl) for 45 s. Successively, 144 ml of 1.25 % sodium cholate solution in water was slowly added to the obtained emulsion: the resulted mixture was further emulsified for 3 min, in an ice bath, at the above-reported amplitude (Supplementary Figures, SI). The BODIPY505-515 (BODIPY)@PNPs were likewise prepared, except that the dye was dissolved into the chloroform oil phase together with the polymer.

After the formation of the final emulsion, chloroform was entirely evaporated under reduced pressure and the resulting particles washed and concentrated by using centrifugal filter devices (Amicon Ultra, Ultracell membrane with 100,000 NMWL, Millipore) to a final volume of 2 ml and finally filtered by using a syringe filters of nylon (13 mm, 0.22 μm, Nazionale, Italy). Cis-Pt@PNPs or BODIPY@PNPs were diluted in H2O and a solution of N-hydroxysulfosuccinimide 2.3 mM (4.3 ml), and a solution of 1-ethyl-3-(3-dimethylaminopropyl)carbodiimide 0.28 M (1.8 ml) (EDC) was added. The reaction was carried out at room temperature (RT) for 10 min, then 102 pmol of CL4 aptamer or SCR, properly activated and dissolved in 1 ml of water, were added and left to react for 24 h. For the preparation of Cyanine7(Cy7)-labelled PNPs (Cy7@PNPs), blank empty PNPs were prepared and, right before the aptamer addition, a solution of 72 µl of Cy7 (bearing a free amino group, Lumiprobe GmbH, Hannover, Germany) in H2O was added for reaching the final Cy7 concentration of 100 nmol/ml.

### Cell lines and culture conditions

Human TNBC MDA-MB-231 and BT-549 cells were purchased from the American Type Culture Collection (ATCC, Manassas, VA) and were grown in Roswell Park Memorial Institute-1640 medium (RPMI-1640, Sigma-Aldrich) supplemented with 10 % fetal bovine serum (Sigma-Aldrich) as previously reported [33]. MDA-MB-231 cells depleted from EGFR by a CRISPR/Cas9 approach (MDA-MB-231 EGFR-KO) were generated by using the CRISPR/Cas9 KO Plasmid and HDR Plasmid Transfection (Santa Cruz Biotechnology, Santa Cruz, CA) following provider’s instruction. All cells were maintained in 5 % CO_2_ atmosphere at 37 °C.

### Confocal Microscopy

MDA-MB-231, BT-549 or MDA-MB-231 EGFR-KO cells (1.0 × 10^5^ cells/well in 24-well), previously seeded on a coverslip for 24 h, were incubated with BODIPY@PNPs-CL4 or BODIPY@PNPs-SCR, diluted at 2 µM dye concentration in culture medium, from 30 up to 60 min. After three washes with Dulbecco’s phosphate-buffered saline (DPBS), cells were fixed with 4 % paraformaldehyde in DPBS for 20 min. Then, cells were incubated with Wheat Germ Agglutinin Alexa Fluor 647-conjugated (WGA-647, Invitrogen, Carlsbad, CA), for 20 min at RT and washed three times with DPBS. Finally, nuclei were stained with 1.5 µM 4′,6-Diamidino-2-phenylindole (DAPI, D9542, Sigma-Aldrich) in DPBS for 5 min and coverslips were mounted with glycerol/DPBS. Samples were visualized by Zeiss LSM 700 META confocal microscopy equipped with a Plan-Apochromat 63x/1.4 Oil DIC objective.

### Immunoblot

Cell and tumor lysates preparation and immunoblot analyses were performed as previously reported [34, 35]. Filters were probed with the indicated primary antibodies: anti-EGFR, anti-phospho-44/42 MAPK (extracellular signal-regulated kinase 1/2, ERK1/2, indicated as pERK1/2), anti-caspase 3 and anti-vinculin (Cell Signaling Technology Inc., Danvers, MA), anti-ERK1 (C-16) (Santa Cruz Biotechnology, Santa Cruz, CA), anti-phospho-histone H2A.X (Ser139, indicated as γH2AX), clone JBW301 (Upstate Biotechnology, Inc., Lake Placid, NY).

Densitometric analysis was performed on at least two different expositions to assure the linearity of each acquisition using ImageJ (v1.46r). Blots shown are representative of at least three independent experiments.

### Cell viability

Viability of MDA-MB-231, BT-549 or MDA-MB-231 EGFR-KO cells (5.0 × 10^3^ cells/well, 96-well plates) treated at the indicated concentrations for indicated periods with free Cis-Pt, Cis-Pt@PNPs, Cis-Pt@PNPs-SCR or Cis-Pt@PNPs-CL4, diluted in cell culture medium, was assessed by CellTiter 96 AQueous One Solution Cell Proliferation Assay (Promega BioSciences Inc., San Luis Obispo, CA) according to the manufacturer’s protocol. Data about cell viability, determined as a percentage of mock-treated cells, were plotted in GraphPad Prism v.8.4.3 to draw dose-response curve and to determine the IC50. Basal toxicity of unloaded PNPs was assessed by cell viability assays on MDA-MB-231 cells after 72 h of treatment with PNPs-SCR or PNPs-CL4 (0.1, 0.5 and 1 mg/ml dry matter concentration).

### Clonogenic Assay

MDA-MB-231 and MDA-MB-231 EGFR-KO cells (200 cells/well, six-well plates) were grown for 21 days, refreshing culture medium once a week. After washes with DPBS, cells were fixed and stained with 0.1 % crystal violet in 25 % methanol. Following 20 min at RT, culture dishes were washed with DPBS and colonies were photographed. 1 % Sodium Dodecyl Sulphate was added on the cells perfectly washed, in order to induce crystal violet dissolution. Absorbance was recorded at 490 nm by a 96-well-plate ELISA reader.

### Cell Apoptosis by flow cytometry analysis

One day after plating (3.5 × 10^5^ cells/well, six-well plates), MDA-MB-231 cells were treated with Cis-Pt@PNPs-SCR or Cis-Pt@PNPs-CL4 (Cis-Pt-concentration, 2 µM) or 10 µM Cis-Pt. Mock-treated cells (Mock) served as a control. After 72 h, cells were detached from culture plates with 0.02 % EDTA (Invitrogen) and stained with Annexin-V and propidium iodide (PI) by using Annexin-V-FLUOS staining Kit (Roche Diagnostics GmbH - Mannheim, Germany), accordingly to provider’s instruction. Cells were suspended in 500 µl incubation buffer and analyzed by flow cytometry (BD Accuri™ C6).

### Tumor targeting with Cy7@PNPs

All experimental procedures described were performed under general anesthesia with 2 % isoflurane in 100 % oxygen at 0.8 l/min. To minimize autofluorescence, the mice were maintained on a diet with a purified, alfalfa-free rodent chow for 15 days before fluorescence imaging [36]. Subcutaneous TNBC xenograft models were realized as previously described [36]. Briefly, 10 × 10^6^ MDA-MB-231 target cells or MDA-MB-231 EGFR-KO non-target cells were re-suspended in 0.1 ml of 1:1 mixture of physiological saline solution and Matrigel (BD Biosciences, Franklin Lakes, NJ) and injected subcutaneously (*s.c.*) into the right flank of six-week-old female Hsd: Athymic Nude-*Foxn1*^nu^ mice weighing approximately 18–20 g (Envigo, Udine Italy). Mice bearing established tumors, approximately 100 mm^3^ [volume = 0.5 × long diameter × (short diameter)^2^], were randomized into groups of three animals per group. Cy7@PNPs, Cy7@PNPs-CL4, Cy7@PNPs-SCR (5 nmol of dye/100 µl of injection) were injected into the lateral tail vein of mice maintained under isoflurane anesthesia using a catheter to improve the likelihood of a successful injection. Fluorescence Reflectance Imaging (FRI) acquisition was performed on the 750 nm channel before and at specified time points, i.e., 30 min, 1, 3 and 24 h after intravenous injection using an FMT 4000 imaging system (PerkinElmer Inc., Waltham, MA, USA), as previously reported [36, 37]. The mice, under general anesthesia, were placed in left lateral recumbency into the imaging cassette, with the tumor xenograft uppermost towards the acquisition device. The imaging cassette was adjusted at 15 or 17 mm of depth, according to the animals’ size. During the imaging session, anesthesia was titrated to 1.5 % isoflurane to obtain the immobility of the subject, and the subject temperature was kept within physiological limits with an integrated system. At 24 h, animals were euthanized and the following organs were harvested: the tumor, liver, spleen, kidneys, lungs and the heart; the gastrocnemius muscle was harvested and acquired for normalization, as well. These organs were acquired in FRI on the 750 nm channel.

Images were reconstructed and analyzed with the proprietary software (TrueQuant© v. 3.1.3, PerkinElmer Inc.). An ovoidal region of interest was placed on the tumor area, i.e., the right flank/thigh, and the signal intensity in counts/energy was recorded on an electronic spreadsheet (Microsoft Excel© for MAC v. 16.48, Microsoft Corporation, Redmond, WA, USA). Signal intensities (SI) were time- and background-corrected by applying the formula:

$$\mathrm{Corrected}\;\mathrm{SI}\;=\;(\mathrm{SI}\;\mathrm{post}-\mathrm{SI}\;\mathrm{pre})/(\mathrm{SI}\;\mathrm{pre})$$

where SI post is the SI at each specified time point, SI pre is the SI before injection. *Ex vivo* acquisition of the aforementioned organs was normalized with respect to the muscle SI.

### Inhibition of tumor growth with Cis-Pt@PNPs

Mice bearing subcutaneous MDA-MB-231 xenografts (approximately 120 mm^3^ tumor volume) were randomly divided into four groups (five animals per group) as follows: group 1, Cis-Pt@PNPs-CL4-treated; group 2, Cis-Pt@PNPs-SCR-treated; group 3, Cis-Pt-treated; group 4, PBS-treated (indicated as Ctrl). Mice of groups 1 and 2 were treated by caudal vein injection with the respective nanoformulation (0.6 mg Cis-Pt payload/kg mean body-weight in 100 µl injection), whereas mice of group 3 were *iv* injected with free Cis-Pt (0.6 mg/kg in 100 µl injection). All treatments were made at day 0, 2, 5, 7, 9, 13, 16, 19, 21 and day 0 was defined as the first day of injections. The dose regimen was chosen taking into account previous Cis-Pt-response studies in MDA-MB-231 xenografts [38, 39] and our previous experience with aptamer-conjugated and drug-loaded PNPs in other tumors [31]. The long and short diameters of the tumors were measured using slide calipers up to 2 days after the last administration (day 23) [volume = 0.5 × long diameter × (short diameter)^2^] and the body weight was also measured. At day 23, mice were euthanized.

After tumor growth experiments, mice were sacrificed and tumors from each animal were excised and frozen, using liquid nitrogen, for protein lysis and immunoblot analyses.

### Statistical analysis

All statistical values were defined using GraphPad Prism version 8.4.3 and comparisons between multiple groups were performed using a one-way analysis of variance (ANOVA) followed by Tukey’s multiple comparison test. *P* value < 0.05 was considered significant for all analyses.

For the tumor targeting analyses, corrected SI were tested for normality with the Shapiro-Wilk’s test and then compared between groups with a two-way repeated measures analysis of variance (RM-ANOVA) mixed-effect model with the Geisser-Greenhouse correction for sphericity, looking for both within and between groups significant differences. *Post hoc*, a Bonferroni test for multiple comparisons was applied within groups, and a Fisher’s least significant difference test between groups. The mean normalized SI of *ex vivo* organs and tumors were compared between groups with the Kruskal-Wallis test, and the Dunn’s test for multiple comparisons.

## Results

### Synthesis and characterization of the multifunctional nanovectors

Various PNPs systems were prepared with the aim of demonstrating different points: selective cell internalization, tumor targeting and biodistribution, *in vitro* cytotoxicity, anti-tumor effect both *in vivo* and *ex vivo*. The common base for all the samples was the PLGA-b-PEG copolymer, able to generate stable nanoparticles’ emulsions. Thanks both to a very versatile formulation procedure, called water-in-oil-in-water double-emulsion, and to the lipo and hydro blocks of the polymer, it is easy to encapsulate molecules whether inside the ‘oil’ moiety, as the lipophilic dye BODIPY for visualization purposes *in vitro*, or inside the inner ‘hydro’ portion, as the Cis-Pt to assess the anti-tumor effect *in* and *ex vivo*. For the selective targeting of EGFR, the so differently loaded PNPs have been further conjugated onto the external surface with CL4 aptamer, or its scrambled sequence (SCR) with no affinity for EGFR [22–24, 31, 34] as control. Eventually, for following the nanovectors biodistribution *in vivo*, together with the tumor targeting, a near infrared Cy7 was covalently labelled onto the PNPs surface (see Methods and Supplementary Information for details, Figures S1, S2, S3).

**Fig. 1:**
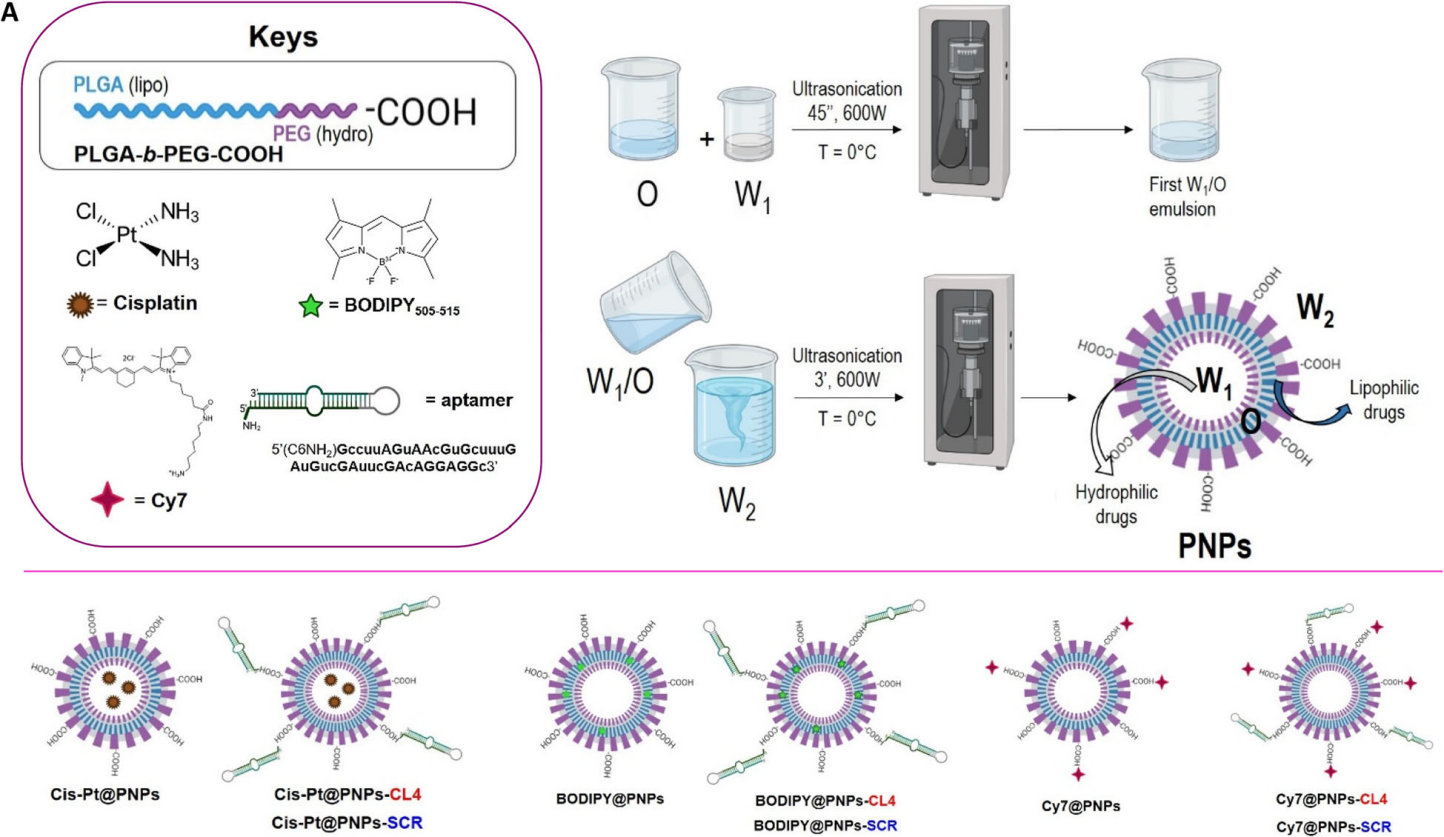
Schematic representation of the Water-in-Oil-in-Water protocol for the obtainment of the final PNP. Encapsulated (Cis-Pt and BODIPY) and covalently labelled (Cy7 and CL4/SCR) molecules were used for multifunctional nanovectors loading/decoration. Above all, the PLGA-*b*-PEG-COOH schematic structure consisting in the lipo and hydro fragments

For a better understanding of the composition and final destination of the nanovectors, a schematic figure representing the samples is provided (Fig. 1).

The PNPs fabrication starts with the copolymer PLGA-b-PEG-COOH synthesis, following a procedure previously reported [17]. The nanoparticles emulsion is then made by means of the water-in-oil-in-water double-emulsion sonication method [40]; we entrapped Cis-Pt in the hydrophilic core of PNPs, obtaining a final water dispersible formulation. After purification of the obtained Cis-Pt@PNPs, the conjugation of the amino-terminated CL4 (or SCR) was performed *via* EDC chemistry, by exploiting the superficial residual carboxylic groups onto the PNPs surface, derived from the PEG chains. In order to demonstrate that there is an effective correspondence between the results obtained and the presence of a specific aptamer onto the surface of the nanoparticles, Cis-Pt@PNPs were also conjugated with SCR and therefore used as a negative control (indicated as Cis-Pt@PNPs-SCR).

Cis-Pt@PNPs-CL4 (or Cis-Pt@PNPs-SCR) were then purified and fully characterized by dynamic light scattering (DLS), which revealed particles with diameter equal to 91.2 ± 1.4 nm, a low polydispersity index (PDI = 0.290), and negative ζ-potential value of − 12.2 mV due to unreacted carboxylic acid groups. Overall, all types of PNPs formulations displayed size ranges suitable for IV administration for long-circulating sustained-release features. Size plays a key role in determining the PNPs’ fate upon administration to the body [41]: a diameter of less than 10 nm results in the removal of NPs by renal filtration, while, on the other hand, PNPs with hydrodynamic radii of 200 nm or more show a higher rate of clearance compared to the ones with lower size range [42]. For an effective penetration, the particle diameter should ideally be 70–200 nm as it will sustain longer circulation time and increased accumulation in the target site. Our nanovectors fully met these requirements.

Cis-Pt concentration was determined to be around 450 µM by Microwave Plasma Atomic Emission Spectrometry (MP-AES), a method for rapid and sensitive determinations of Pt concentration. The overall concentration of Cis-Pt@PNPs-CL4 in solution was measured by gravimetric analysis to be 7 mg/ml.

On the other hand, for performing fluorescence-based experiments (such as cell internalization of nanoparticles by confocal microscopy and tumor targeting and biodistribution *in vivo* by fluorescence reflectance imaging), we fabricated two additional kinds of nanovectors: the BODIPY@PNPs containing the green dye into the PNPs lipophilic portion, and the Cy7@PNPs, in which Cy7 was covalently conjugated to the carboxylic groups onto the nanoparticles’ external surface. Both the systems were also labelled with CL4 or SCR for targeting purposes, purified and completely characterized (see Supplementary Information, Figures S2 and S3). Table 1 exhibits the various properties of fabricated PNPs formulations.

**Table 1:** Properties of polymeric nanoparticles

PNP formulation	PNPs characteristics				
	**Average size (nm)**	**PDI**	**Zeta potential (mV)**	**Drug/Dye concentration**	**Dry matter amount (mg/ml)**
Cis-Pt@PNPs	90.3 ± 0.3	0.254	-21.4	460.78 µM (0.138 µg/µl)	7
Cis-Pt@PNPs-CL4	91.1 ± 1.4	0.290	-12.2	422.70 µM (0.127 µg/µl)	7
Cis-Pt@PNPs-SCR	118.5 ± 20	0.290	-11.1	455.18 µM (0.136 µg/µl)	7
BODIPY@PNPs	97.6 ± 0.3	0.142	0.08	166 µM	8
BODIPY@PNPs-CL4	131.9 ± 0.2	0.185	-17.9	13.6 µM	5
BODIPY@PNPs-SCR	142.2 ± 1.5	0.185	-23.4	9.7 µM	5
Cy7@PNPs	90.5 ± 0.5	0.218	-24.9	104.16 µM	4
Cy7@PNPs-CL4	107.9 ± 0.3	0.350	-20.6	106.8 µM	6
Cy7@PNPs-SCR	104.2 ± 2.7	0.350	-20.6	105.96 µM	6

### ***In vitro*** targeting by aptamer-conjugated nanovectors

**Fig. 2:**
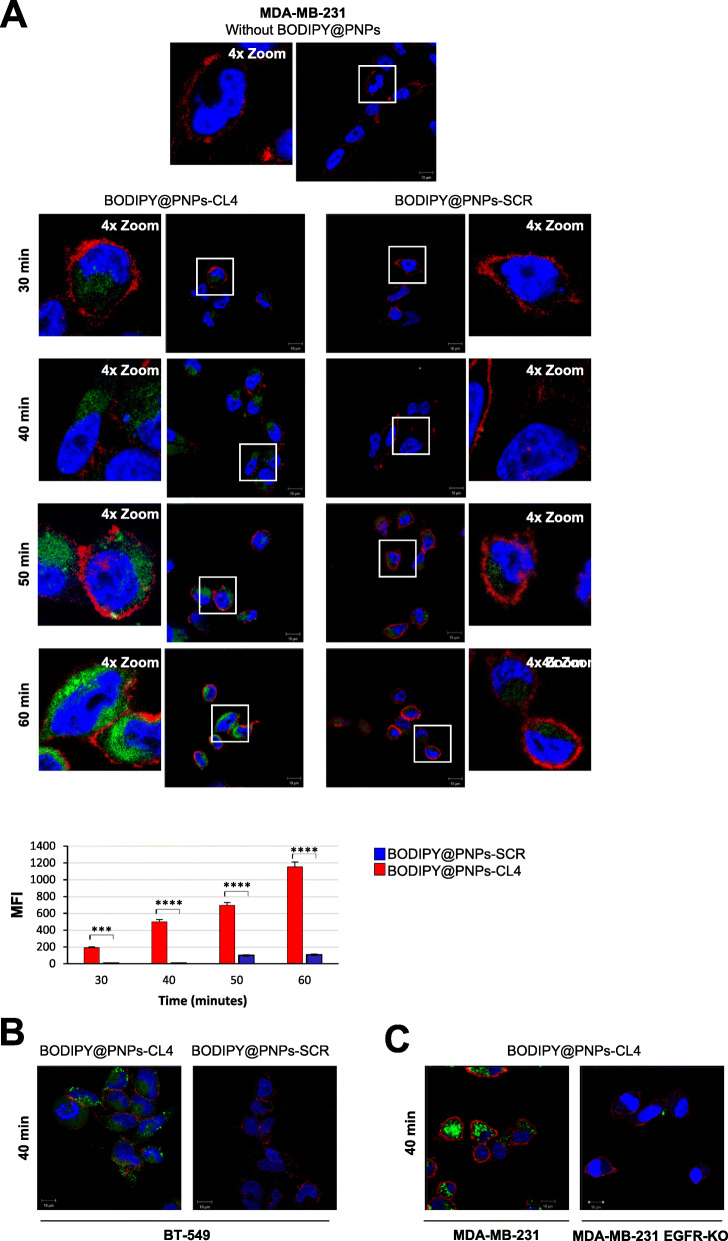
Selective cell uptake of BODIPY@PNPs-CL4 compared to BODIPY@PNPs-SCR. (**a**) *Upper*, Representative confocal images of MDA-MB-231 cells left untreated or treated with BODIPY@PNPs-CL4 or BODIPY@PNPs-SCR at 37 °C for different periods (from 30 to 60 min). After washing and fixation, cells were labelled with WGA (red) to visualize cell membrane and with DAPI (blue) to stain nuclei. BODIPY@PNPs are displayed in green. White squares indicate the area shown in insets in a magnified view obtained using Image J software. *Lower*, mean fluorescence intensity (MFI) was evaluated by Zeiss software on a minimum of 50 cells for each sample (*n* = 3). ****P < 0.0001; ***P < 0.001. (**b**) Representative confocal images of BT-549 cells treated with BODIPY@PNPs-CL4 or BODIPY@PNPs-SCR at 37 °C for 40 min. (**c**) Representative confocal images of MDA-MB-231 and MDA-MB-231 EGFR-KO cells treated with BODIPY@PNPs-CL4 at 37 °C for 40 min. (**a-c**) Magnification 63×, 1.0× digital zoom, scale bar = 10 μm. All digital images were captured at the same setting to allow direct comparison of staining patterns

To assess whether the CL4 aptamer is able to specifically target the nanovectors to EGFR-positive TNBC cells and enhance their intracellular uptake, we exploited the BODIPY-loaded PNPs decorated with CL4 (BODIPY@PNPs-CL4) or SCR (BODIPY@PNPs-SCR) as a negative control. MDA-MB-231 cells, which represent a well-established model for aggressive TNBC [43–45] and express abundant levels of EGFR [24, 33] (Figure S4A), were incubated with fluorescent nanovectors for different times (from 30 to 60 min) at 37 °C and visualized by confocal microscopy (Fig. 2a). As shown, the signal associated with BODIPY@PNPs-CL4 was clearly visible in the cytoplasm at 30 min and further increased in a time-dependent manner. Conversely, a very weak signal was detected with SCR-decorated nanovectors only starting at 50 min, which remains unchanged for up to 60 min of incubation, resulting about 10-fold lower than the signal associated with CL4-targeted nanovectors. Furthermore, in agreement with the high efficacy of aptamer targeting toward EGFR-positive BT-549 TNBC cells ([24] and Figure S4A), BODIPY@PNPs-CL4, but not BODIPY@PNPs-SCR, were internalized into these cells as well (Fig. 2b). This indicates that the nanoparticle carriers’ parameters, including composition, size, charge and shape, do not affect the dynamics of CL4 aptamer-receptor interactions on membranes of both MDA-MB-231 and BT-549 cancer cell lines.

Finally, further confirming the specificity of the EGFR aptamer [19–21, 24], BODIPY@PNPs-CL4 could distinguish MDA-MB-231 cells from EGFR-depleted MDA-MB-231 cells obtained by a CRISPR/Cas9 approach (Fig. 2c and Figure S4A), demonstrating the excellent selective internalization of CL4-conjugated nanoparticles.

Altogether, these data clearly indicate that the CL4 aptamer specifically delivers PNPs to EGFR-positive TNBC cells, strongly enhancing their intracellular uptake.

### ***In vitro*** cytotoxicity of aptamer-conjugated and cisplatin-loaded nanovectors

**Fig. 3:**
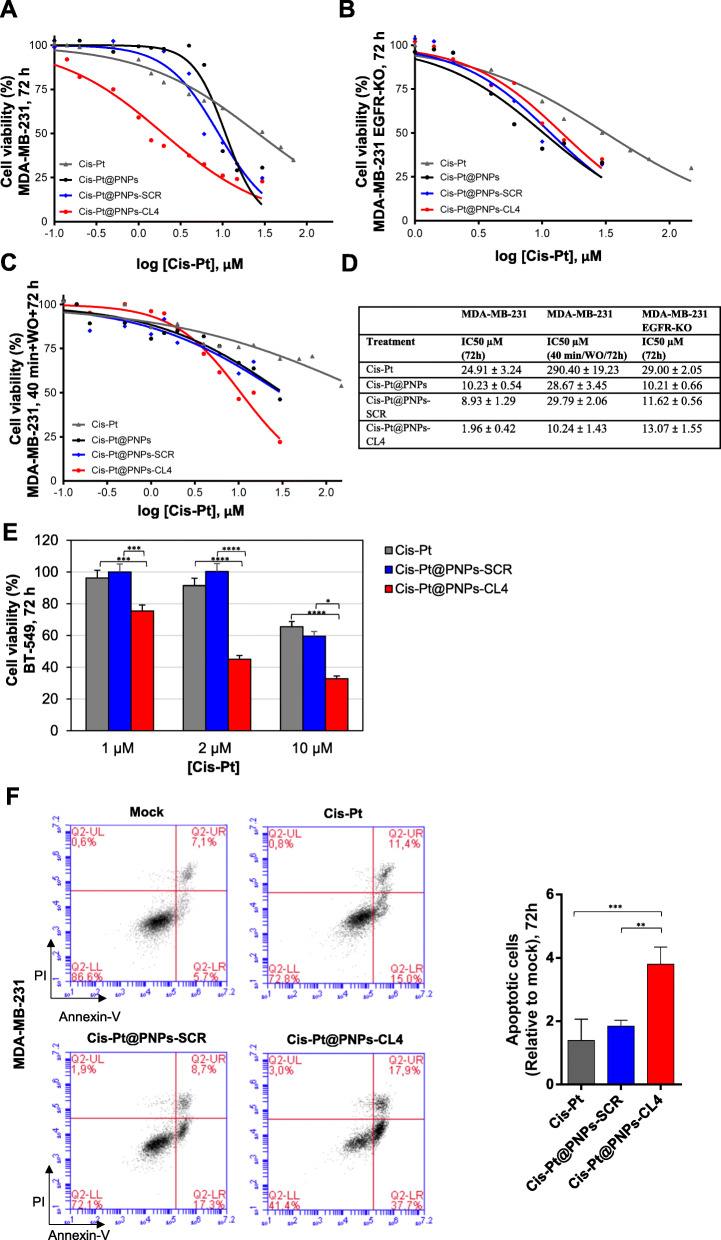
Cis-Pt@PNPs-CL4 are more cytotoxic than free Cis-Pt and untargeted Cis-Pt-loaded nanovectors. (**a-c**) Representative IC50 curves for free Cis-Pt, Cis-Pt@PNPs, Cis-Pt@PNPs-SCR and Cis-Pt@PNPs-CL4 on MDA-MB-231 and MDA-MB-231 EGFR-KO. Cells were incubated with increasing concentration of free Cis-Pt, Cis-Pt@PNPs, Cis-Pt@PNPs-SCR or Cis-Pt@PNPs-CL4 (from 0.1 to 30 µM Cis-Pt, free or encapsulated in nanoparticles). After continuous 72 h-incubation (**a, b**) and after 40 min incubation followed by washes (WO = washout) and 72 h recovery (**c**), cell viability for each sample was determined and expressed as percent of viable treated cells with respect to mock-treated controls. Data were plotted in GraphPad Prism v.8.4.3 software to draw dose-response curve and to calculate IC50 values (**d**)**.** IC50 was estimated on the basis of at least three different experiments. (**e**) BT-549 cells were incubated with Cis-Pt, Cis-Pt@PNPs-SCR or Cis-Pt@PNPs-CL4 at the indicated concentration of Cis-Pt for 72 h and cell viability for each sample was analyzed and expressed as percent of viable treated cells with respect to mock-treated controls. Each determination represents the average of three individual experiments and error bars represent SD. ^****^P < 0.0001; ^***^P < 0.001; ^*^P < 0.05. (**f**) The apoptosis of MDA-MB-231 cells receiving 72-h treatment with Cis-Pt, Cis-Pt@PNPs-CL4 or Cis-Pt@PNPs-SCR was measured by Annexin-V/PI staining and flow cytometric analysis. The cell apoptosis data are shown on the right histograms. Values are shown relative to mock treatment, arbitrarily set to 1 (*n* = 3). ^***^P < 0.001, ^**^P < 0.01

In order to assess the ability of CL4 aptamer-conjugated and Cis-Pt-loaded PNPs to specifically kill EGFR-positive cells, MDA-MB-231 cells were incubated for 72 h with increasing concentrations of the drug, both free and entrapped in PNPs, ranging from 0.1 to 30 µM, and cell viability was determined using an MTT assay. As shown (Fig. 3a and d), Cis-Pt loaded in the nanoparticles, either unconjugated or conjugated with SCR, was almost 2.5-fold more cytotoxic than free drug (P < 0.01, both), with half maximal inhibitory concentration (IC50) values of 10.23 ± 0.54 µM and 8.93 ± 1.29 µM for Cis-Pt@PNPs and Cis-Pt@PNPs-SCR, respectively, and 24.91 ± 3.24 µM for free Cis-Pt. Notably, the conjugation of CL4 aptamer significantly increased the cytotoxicity of the Cis-Pt-loaded nanoparticles that displayed an IC50 of 1.96 ± 0.42, thus approximately 12-fold lower than that of free drug (P < 0.0001) and 5-fold lower than that of Cis-Pt@PNPs-SCR or Cis-Pt@PNPs (P < 0.05). Very limited basal toxicity of unloaded PNPs conjugated to either CL4 or SCR aptamers was observed up to 1 mg/ml (Figure S5), the maximal carrier concentration used in cytotoxicity studies with Cis-Pt loading. These data confirm that PNPs are extremely safe and atoxic [31]. Furthermore, as expected based on the high specificity of the EGFR aptamer, superimposable cell viability curves were obtained upon incubation of CL4-targeted PNPs and untargeted PNPs, both unconjugated and decorated with SCR, onto MDA-MB-231 EGFR-KO cells (Fig. 3b and d). Interestingly, these cells had a response to Cis-Pt treatment comparable to that of parental cells (Fig. 3d), but a higher proliferative potential, as assessed by clonogenic growth rate analysis (Figure S4B), thus suggesting the occurrence of compensatory pathways to EGFR silencing. Then, we performed a short 40-min incubation with Cis-Pt, free or loaded into PNPs, followed by washing and 3 days recovery period. Under these experimental conditions, viability of MDA-MB-231 cells was much less affected by free Cis-Pt (IC50 value of 290.40 ± 19.23 µM) with respect to the 72-h continuous exposure to the drug (Fig. 3c and d). Importantly, IC50 of free Cis-Pt decreased of about 28-fold (P < 0.0001) when the drug was incapsulated into the CL4-equipped PNPs (Cis-Pt@PNPs-CL4, IC50 10.24 ± 1.43), thus indicating that Cis-Pt@PNPs-CL4 strongly improved drug accumulation in cancer cells. Moreover, Cis-Pt@PNPs-CL4 were almost 3-fold more effective than either Cis-Pt@PNPs or Cis-Pt@PNPs-SCR (P < 0.05) (Fig. 3c and d), further confirming the rapid internalization of EGFR aptamer-driven nanoparticles and more efficient killing of TNBC cells than under passive delivery conditions.

Next, we verified that Cis-Pt@PNPs-CL4 are also more efficient in reducing viability of BT-549 cells than free Cis-Pt and control Cis-Pt@PNPs-SCR nanovectors (Fig. 3e). Indeed, even at low drug concentration, at which control nanovectors and free drug did not affect cell viability, the targeted nanovectors caused significant cancer cells killing (approximately 50 % inhibition at 2 µM-concentration, P < 0.0001).

Moreover, in order to evaluate whether the 72-h treatment of MDA-MB-231 cells with free Cis-Pt (10 µM) and Cis-Pt-loaded nanovectors decorated either with CL4 or SCR (Cis-Pt-concentration, 2 µM) induced apoptosis, the cell death was measured by flow cytometry analysis after Annexin-V/PI dual staining. As shown, (Fig. 3f), while free Cis-Pt and Cis-Pt@PNPs-SCR had no significant effect, Cis-Pt@PNPs-CL4 treatment significantly increased the apoptotic cell proportion from ~ 13 % (Mock treatment) to ~ 56 % (Cis-Pt@PNPs-CL4).

Overall, these data demonstrate the ability of Cis-Pt@PNPs-CL4 to efficiently concentrate Cis-Pt in EGFR-positive TNBC cells and kill them, furthermore by discriminating target cells from non-target cells that do not express EGFR.

### ***In vivo*** tumor targeting by aptamer-conjugated nanovectors

**Fig. 4:**
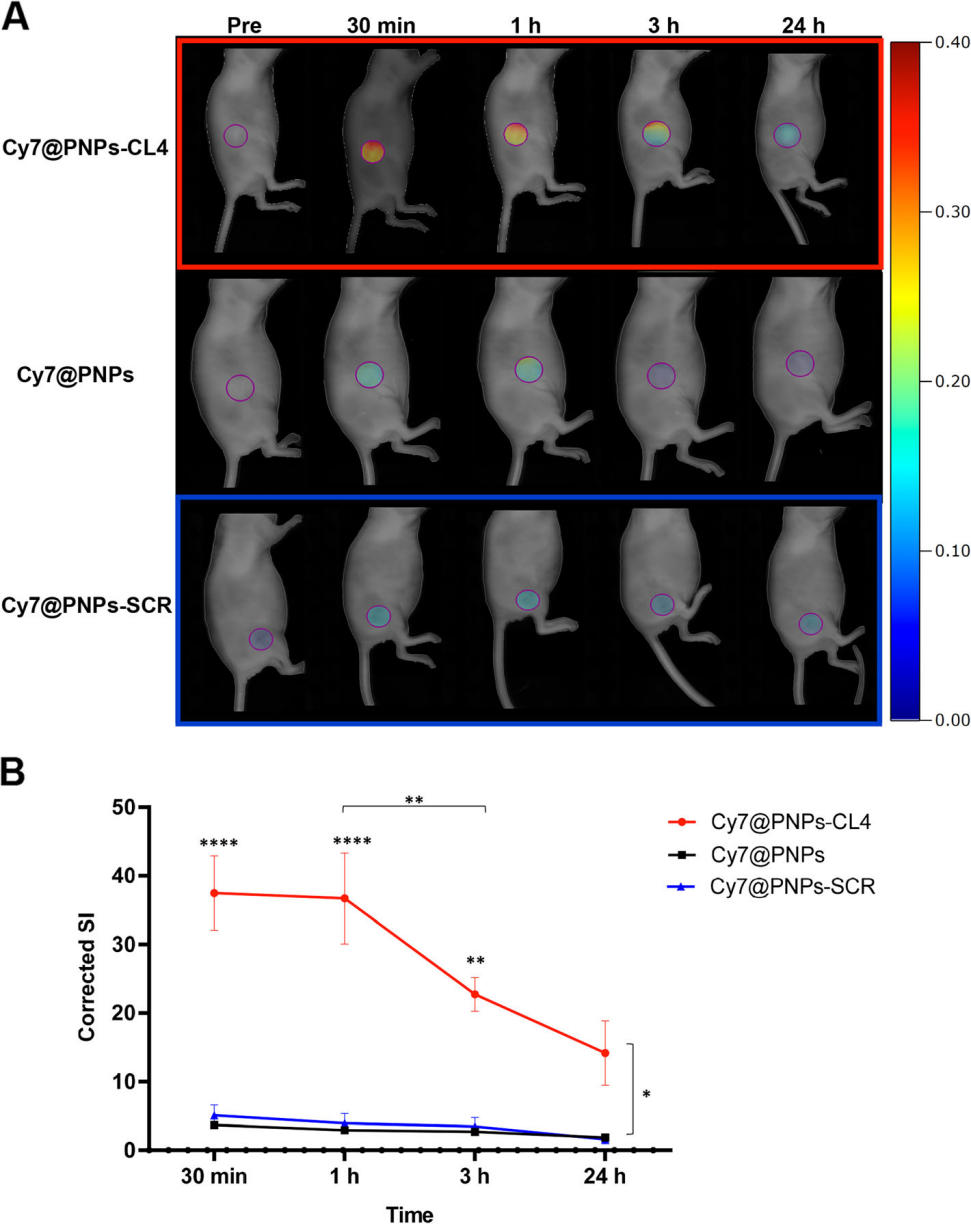
Selective tumor targeting of Cy7@PNPs-CL4 compared to Cy7@PNPs-SCR and Cy7@PNPs.** (a)** Nude mice bearing subcutaneous MDA-MB-231 xenografts (three animals/group) were *i.v.* injected with Cy7@PNPs-CL4, Cy7@PNPs or Cy7@PNPs-SCR (5 nmol Cy7/100 µl) and analyzed by *in vivo* FRI imaging at the indicated time points (i.e., Pre: before injection, 30 min, 1 h, 3 and 24 h acquisitions). Representative images for Cy7@PNPs-CL4, Cy7@PNPs-SCR and Cy7@PNPs injected mice are shown. The scale bar is in arbitrary units and is a colorimetric representation of the minimum and maximum signals; all the depicted images are reconstructed with the same scale. **(b)** Graphical representation of *in vivo* corrected Signal Intensity (SI) at the selected time points in the three groups. ^****^P < 0.0001, ^**^P < 0.01, ^*^P < 0.05, relative to 24 h acquisition if not differently indicated by connecting lanes

To evaluate the *in vivo* tumor targeting efficiency of CL4-conjugated nanovectors, Cy7@PNPs-CL4 and its non-targeting variants, Cy7@PNPs-SCR and unconjugated Cy7@PNPs, were administered intravenously *via* tail vein (Cy7-concentration, 5 nmol/100 µl injection) into MDA-MB-231 tumor bearing nude mice and non-invasive imaging was performed over 24 h by FRI. As shown (Fig. 4a), Cy7@PNPs-CL4-associated fluorescence signal was readily detected in tumors as early as 30 min after injection, persisted at 1 h and then steadily decreased as the time was prolonged up to 24 h, but nonetheless remained higher than both unconjugated Cy7@PNPs and SCR-conjugated PNPs, which were poorly detected in tumors at all experimental time-points. Figure 4b showed that, within groups, only in Cy7@PNPs-CL4 treated mice the corrected signal intensity (SI) at 30 min, 1 and 3 h was significantly higher than 24 h (*P* < 0.0001, *P* < 0.0001 and *P* = 0.0086, respectively) and at 30 min and 1 h was significantly higher than 3 h (*P* < 0.01). No other significant differences were detected in the other groups. Moreover, between groups, only Cy7@PNPs-CL4 treated mice had a significantly higher corrected SI compared to both Cy7@PNPs and Cy7@PNPs-SCR treated mice at 30 min (*P* = 0.022 and P = 0.036, respectively), at 1 h (*P* = 0.029 and *P* = 0.041, respectively), at 3 h (*P* = 0.037, both) and at 24 h (*P* = 0.019 and *P* = 0.026, respectively).

**Fig. 5:**
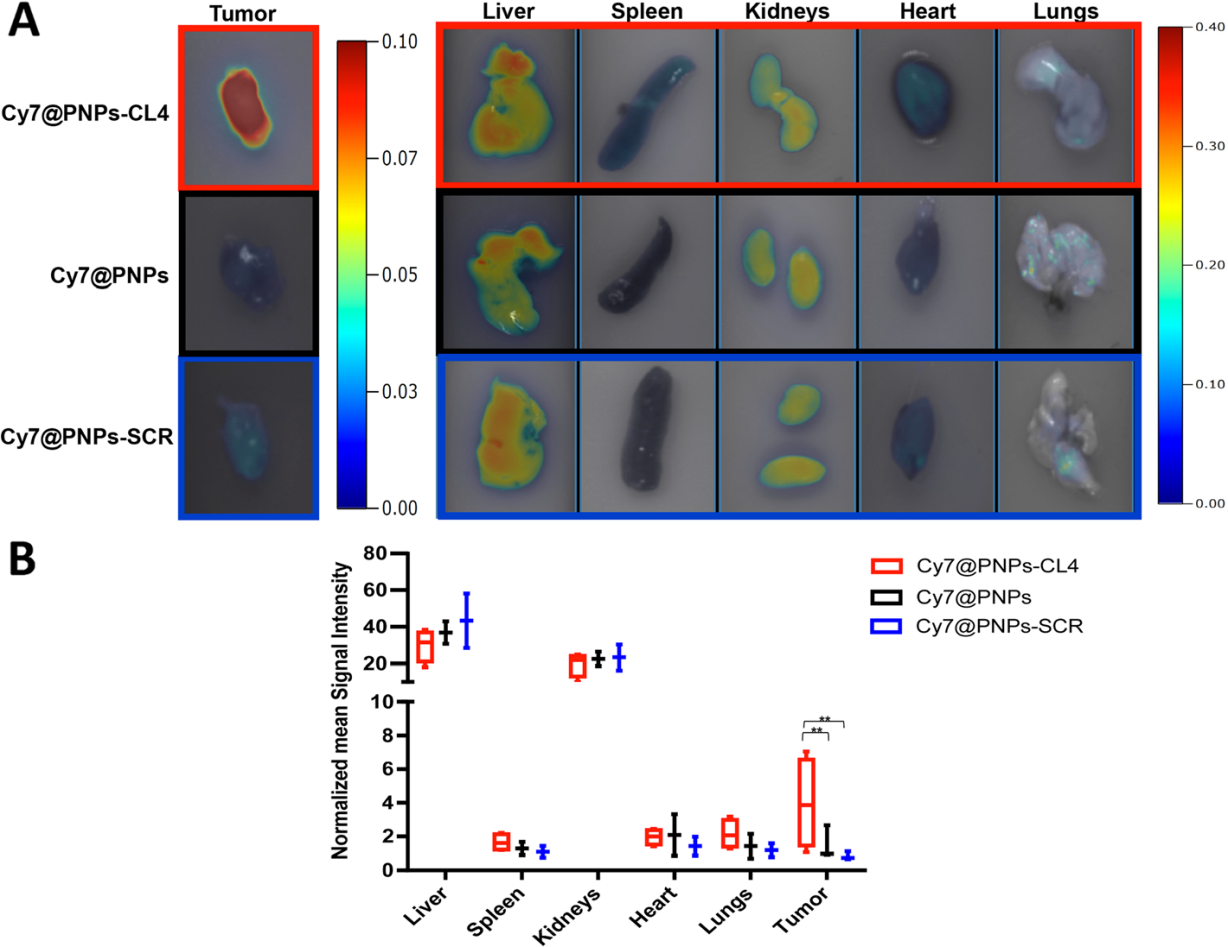
*Ex vivo* FRI analysis. **(a)***Ex vivo* FRI of representative tumors (left) and different organs (right) explanted from mice 24 h post-injection with Cy7@PNPs-CL4, Cy7@PNPs or Cy7@PNPs-SCR treatment groups. The scale bars are in arbitrary units and are a colorimetric representation of the minimum and maximum signals; for comparing the three different treatments, all the depicted images are reconstructed with the same scale. **(b)** Graphical representation of the normalized mean FRI Signal Intensity ± DS (n = 3) of tumors and organs in the three groups. ^**^P ≤ 0.01 relative to both Cy7@PNPs and Cy7@PNPs-SCR

The main organs and tumors of the mice were harvested 24 h after PNPs injection for evaluation of biodistribution by *ex vivo* FRI imaging (Fig. 5). Consistent with *in vivo* imaging results, Cy7@PNPs-CL4 showed greater intra-tumor accumulation than unconjugated PNPs and SCR-conjugated PNPs (Fig. 5a). Indeed, the normalized mean SI of tumors was significantly different between groups, being in Cy7@PNPs-CL4 group approximately 4.7- and 2.6-fold higher than in Cy7@PNPs-SCR and Cy7@PNPs groups, respectively (P = 0.01 both; Fig. 5b). Conversely, there was no significant difference in normalized mean SI between groups for liver, spleen, kidneys, heart, and lungs (Fig. 5a and b). As expected, a high fluorescence signal was observed in the liver and kidneys of all groups accordingly to the detoxification function and the elimination routes for biodegradable nanoparticles of liver and kidney, respectively [46], whereas not substantial accumulation was seen in other organs such as lung, heart and spleen (Fig. 5a).

Further, confirming the *in vitro* findings, Cy7@PNPs-CL4 were able to discriminate MDA-MB-231 tumors from MDA-MB-231 EGFR-KO-derived tumors (Figure S6), thus indicating the targeting selectivity of the nanovectors for EGFR-positive tumors.

Taken together, these results clearly demonstrate tumor-specific uptake of Cy7@PNPs-CL4 and thus the feasibility of CL4 aptamer-conjugated PNPs as an efficient delivery vehicle in a targeted manner.

### ***In vivo*** antitumor efficacy of aptamer-conjugated and cisplatin-loaded nanovectors

**Fig. 6:**
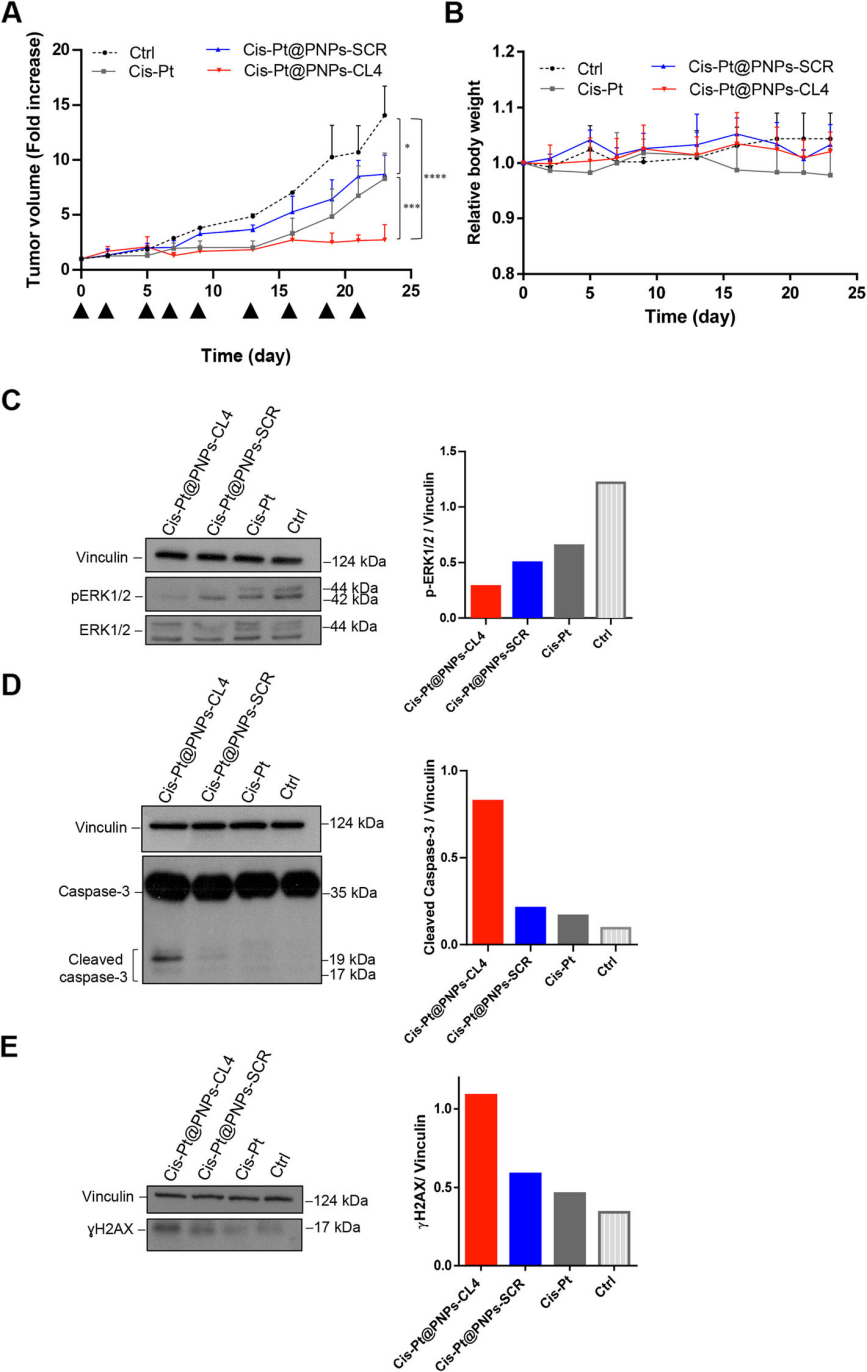
*In vivo* antitumor efficacy of Cis-Pt@PNPs-CL4 compared to free Cis-Pt and Cis-Pt@PNPs-SCR. Mice bearing MDA-MB-231 xenografts were injected intravenously with free Cis-Pt, Cis-Pt@PNPs-CL4 or Cis-Pt@PNPs-SCR (0.6 mg Cis-Pt/kg) at the times indicated by the head arrows. Day 0 marks the start of treatments. Mice treated with DPBS were used as the control group (Ctrl). **(a)** Tumor growth was monitored by calipers over time and experimental raw data (expressed as fold change) were interpolated with no curve fitting or regression analysis. Each point represents the mean ± SD of five mice. ^****^P < 0.0001, ^***^P < 0.001, ^*^P < 0.05. **(b)** Body weight changes in mice of each group at the indicated days. The results represent the means ± SD (*n* = 5). **(c-e)** Immunoblot with anti-pERK1/2 and ERK1/2 **(c)**, anti-caspase-3 **(d)** and anti-γH2AX **(e)** antibodies of pooled lysates from recovered tumors (*n* = 5). Vinculin was used as an internal control. The histograms indicate pERK1/2/vinculin **(c)**, cleaved caspase-3/vinculin **(d)** and γH2AX/vinculin **(e)** ratio of densitometric signals. Depicted results represent one of three typical experiments performed. Molecular weights of indicated proteins are reported

Given the excellent tumor-specific targeting of CL4-PNPs, we next investigated the *in vivo* antitumor efficacy of the Cis-Pt-loaded nanovectors in xenograft models of MDA-MB-231 tumor. To this aim, tumor-bearing mice were injected intravenously with free Cis-Pt, Cis-Pt@PNPs-CL4 or Cis-Pt@PNPs-SCR as control nanovectors at day 0, 2, 5, 7, 9, 13, 16, 19, 21. Mice treated with DPBS served as control (Ctrl). Tumor growth was monitored over time (up to 23 days). In order to appreciate the potential effectiveness of our targeted chemotherapy, Cis-Pt@PNPs-CL4 and untargeted Cis-Pt@PNPs-SCR were administered at 0.6 mg/kg-Cis-Pt concentration and their effects compared with that of the same dosage of free Cis-Pt. This dosage was at least 5-fold lower than the minimum reference dosage of Cis-Pt (repeated *iv* injection of 5 − 3 mg/kg) used to study the antitumor activity in mice bearing MDA-MB-231 subcutaneous xenografts [38.39]. In this experimental condition (suboptimal concentration of the drug), treatments with either free Cis-Pt or Cis-Pt@PNPs-SCR resulted in a slightly but significantly delayed tumor growth compared to Ctrl treatment (P = 0.0015 and P = 0.018, respectively; Fig. 6a). However, they displayed similar response because of no statistical differences between them, indicating that the non-targeted nanoparticle delivery system was not able to significantly improve the free Cis-Pt efficacy. Conversely, the use of CL4-PNPs as a delivery vehicle for Cis-Pt, resulted in a highly significant inhibitory performance on tumor growth compared to free Cis-Pt (*P* = 0.0004) and Cis-Pt@PNPs-SCR (P = 0.0009) (Fig. 6a).

This indicates that the presence of the EGFR aptamer on the PNP surface was able to concentrate the Cis-Pt payload at the tumor site, thereby enhancing the anticancer activity of the drug. Importantly, the treatment was well tolerated *in vivo*, with no significant bodyweight loss and behavioral change in the treated mice throughout the entire study (Fig. 6b).

Next, immunoblot analyses performed on tumor lysates showed a more efficient inhibition of ERK1/2 phosphorylation in the tumors from mice treated with Cis-Pt@PNPs-CL4 than those treated with Cis-Pt@PNPs-SCR or free Cis-Pt (Fig. 6c). Finally, in agreement with *in vitro* findings, the inhibiting effect of Cis-Pt@PNPs-CL4 was accompanied by a strong activation of caspase-3, a hallmark of apoptosis (Fig. 6d), and accumulation of γH2AX protein, which is a marker of DNA double-strand breaks (Fig. 6e), thus confirming the better efficacy of Cis-Pt when delivered by the EGFR aptamer-conjugated nanovectors.

## Discussion

Targeted delivery of anticancer agents demonstrates clear advantages over the non-targeted delivery of therapeutics. The high affinity and specificity for the target, combined with the absence of toxicity and immunogenicity and adaptable modification procedures, render oligonucleotide aptamers the new generation of targeting agents [47, 48]. Herein, aptamer-based nanosystems have been designed and synthesized to improve cancer treatment efficiency of Cis-Pt, a traditional chemotherapeutic drug that kills cancer cells by damaging their DNA [49].

Cis-Pt is one of the most potent antitumor agents known, widely used against a variety of solid tumors, including lung, head and neck, ovarian, testicular, bladder and colorectal cancers. Even though it has been nearly 40 years since the FDA approved of its use for cancer treatment, there is still a huge effort to fully elucidate how Cis-Pt works in order to improve its antitumor activity and reduce the side effects associated with patient treatment [50]. Cis-Pt does not represent the standard care for TNBCs, however, given the need to apply sequential and combined chemotherapies especially for early-stage cancers, due to the absence of targeted therapies, its use for treating these cancers is currently under intense investigation. Moreover, most TNBCs have a defect in DNA repair [51], thus further increasing interest in the role of platinum drugs and in identifying patients who might benefit from them. Phase-II and Phase-III clinical trials have demonstrated the efficacy of including Cis-Pt and its analogues in neoadjuvant regimens for TNBC [52–57]. Also, patients with metastatic TNBC seem to benefit from platinum treatment [58]. However, results show that, although highly effective in inducing TNBC tumor cell death, major challenges (i.e., drug-resistance occurrence, high toxicity, low stability, drug inactivation, low intracellular uptake, poor intratumor accumulation) exist that still leave open the debate of whether or not TNBC treatment should include platinum salts [59, 60]. In search of efficacious strategies to solve the limitations due to the non-selective biodistribution and poor lipophilicity of Cis-Pt, which limit its uptake in tumor cells, a viable strategy is to use actively targeted nanocarriers to concentrate Cis-Pt specifically at tumor site, while delivering lower absolute doses of the drug, thereby reducing systemic side effects [14, 61]. Here, we addressed this possibility and show for the first time the tumor targeting efficacy and anti-tumor effect of a PLGA-PEG-Cis-Pt nanosystem, equipped with a high specific EGFR aptamer (named CL4), in mice bearing TNBC xenografts. Cis-Pt encapsulation in micelles-like nanovectors or other kind of nanoparticles may result tricky due to its water solubility, which plays against the confinement of the chemotherapeutic in the inner core of nanovectors while favoring its leakage in external media. With an opportune modified emulsion protocol and thanks to the amphiphilicity of PLGA-PEG block copolymer we were able to encapsulate and keep confined inside the nanovectors a water-soluble molecule such as Cis-Pt. The obtain nanoparticles present an inner core composed of hydrophilic PEG portion, a middle bi-layer formed by lipophilic PLGA chains and an outer shell composed again by PEG [62]: this results in the maintenance of carboxylic groups onto the external surface, thus allowing for easily functionalization with targeting and/or fluorescent moieties. The CL4 aptamer is a nuclease-resistant 2′F-Py RNA (39-nucleotide) that we generated by SELEX on chemo-resistant NSCLC cells [21] and matched to EGFR by post-SELEX target identification approaches [19–21, 24]. Its ability to specifically deliver secondary reagents, including chemotherapeutics [29], therapeutic anti-miRNA [26–28] and even antibodies [22, 23] to EGFR-positive cancer cells and tumors has been extensively demonstrated by our and other groups. We now show that the surface modification of PLGA-PEG nanoparticles with CL4 aptamer endows the nanosystem with: (i) excellent cancer cells targeting and internalization capabilities; (ii) rapid uptake (30 min after systemic *i.v*. administration) in EGFR-positive MDA-MB-231-derived tumors, but not in EGFR-negative tumors; (iii) durable tumor retention (at least 24 h) and (iv) no accumulation in healthy organs, i.e., lung and heart.

One intriguing result was the low-intensity signal detected in the spleen. While the liver and kidneys’ prominent role as excretory organs can easily explain their high signal intensities, the absence of almost any signal into the spleen suggests that the PNPs used in our study can escape, at least in part, the reticuloendothelial system (RES). Indeed, it has been well described how phagocytic cells belonging to the RES, i.e. monocytes and macrophages housed primarily in the liver and spleen, rapidly take up nanoparticles when coated by serum proteins and depending on their size and surface characteristics [63–65]. The accumulated nanoparticles can be found up to one month after the administration in both liver and spleen [66]. Hence, the low and high signals in the spleen and liver, respectively, seem to testify that the latter is only a mere route of excretion.

Remarkably, cisplatin encapsulated in aptamer-conjugated nanoparticles caused up to 28- and 5-fold higher cytotoxicity in comparison with free drug and untargeted nanovectors (either unconjugated or conjugated with a scrambled aptamer), respectively, indicating that the targeted delivery system provided improved chemotherapy. We previously demonstrated the ability of CL4 aptamer to bind to (and inhibit) EGFR that is overexpressed, even if at different level, on MDA-MB-231 and BT-549 cells, thus indicating that the receptor is displayed on the surface of both cell lines in a status competent for recognition by the aptamer [24]. Accordingly, the aptamer efficiently delivers Cis-Pt-loaded PNPs to both cell lines discriminating them from EGFR-depleted cells. It remains to be investigated whether a different saturation level of the nanovectors is reached on the two cell lines and how their targeting efficiency may vary with the different receptor density. Consistent with the *in vitro* experiments, Cis-Pt@PNPs-CL4, systemically injected in nude mice bearing MDA-MB-231-derived xenograft tumors at a suboptimal therapeutic dose of Cis-Pt payload (~ 0.6 mg/kg), abolished tumor growth and induced apoptosis with a superior effect respect to free Cis-Pt and Cis-Pt@PNPs-SCR. Interestingly, cisplatin dosage used in mice to inhibit tumor growth (repeated *iv* injection of 3–5 mg/kg) is usually associated to certain degree of toxicity causing severe emaciation or even death of some mice during the study [38, 39, 67]. Conversely, our work shows that while suppressing tumor growth no change in mice body weight was observed upon our active Cis-Pt delivery system, thus showing that it could result in an effective and safe method of achieving selective accumulation of the drug at tumor site in patients’ treatment. From a clinical point of view, we expect that cytotoxicity will be mostly confined to EGFR-overexpressing tumor cells, similarly to what happens for most therapeutic agents against targets preferentially but not exclusively expressed in tumors. However, further investigation in humanized mice models is needed to assess this possibility.

Our group has previously developed nanovectors with the PDGFRβ aptamer conjugated on the PNP outer shell that have been shown to be extremely efficient in delivering a PI3K/mTOR inhibitor to GBM implanted intracranially in mice, across the blood-brain barrier [31]. PDGFRβ is also well expressed on the surface of specific TNBC cells [36]. Further studies will allow us to assess whether the PDGFRβ aptamer, as well as multiple TNBC-targeting aptamers we recently identified [33], may be used in different combinations for delivering drug-loaded nanovectors to TNBC with distinct molecular and/or clinical phenotypes.

## Conclusions

In this study, we successfully constructed PLGA-based nanoparticles loaded with Cis-Pt and conjugated on the outer shell to an EGFR-aptamer as a highly specific targeting agent. The resulting nanovectors proved to be a promising nanoplatform for active tumor-targeted Cis-Pt delivery with the capability of high-yield drug payload, tumor-specific targeting, and improved therapeutic efficacy than untargeted nanovectors and free drug. This novel approach could represent a new strategy for the treatment of EGFR-positive tumors. Interestingly, it may be applied to overcome the intrinsic resistance of TNBCs to EGFR inhibitors by using this receptor as an anchor point for the nanoparticles, decorated with CL4 aptamer, specifically delivering the cytotoxic drug inside them. Thus, a signaling molecule critical for cancer cell survival and proliferation is converted into a cancer vulnerability.

## Supplementary Information

**Additional file 1:**

## Data Availability

All data analyzed during this study are included in this manuscript.

## Abbreviations

2'F-Py: 2’Fluoro-pyrimidines
ATCC: American Type Culture Collection
DAPI: 4′,6-Diamidino- 2-phenylindole
BC: Breast cancer
Cis-Pt: Cisplatin
Cy7: Cyanine 7
DLS: Dynamic light scattering
DMSO: Dimethyl sulfoxide
DPBS: Dulbecco’s phosphate-buffered saline
EDC: 1-ethyl-3-(3-dimethylaminopropyl)carbodiimide
EGFR: Epidermal growth factor receptor ER estrogen receptor
ERK1/2: Extracellular-signal regulated kinase 1/2
FBS: Fetal bovine serum
FRI: Fluorescence Reflectance Imaging
GBM: Glioblastoma
H&E: Hematoxylin and eosin
HER2/ErbB2: Epidermal growth factor receptor 2
MFI: Mean fluorescence intensity
MP-AES: Microwave Plasma Atomic Emission Spectrometry
NSCLC: Non-small-cell lung cancer
PDI: Polydispersity index
PI: Propidium iodide
PLGA: Poly lactic-co-glycolic acid
PLGA-b-PEG: PLGA-block-polyethylene glycol
PNPs: Polymeric nanoparticles
PR: Progesterone receptor
RPMI-1640: Roswell Park Memorial Institute-1640 medium
RT: Room temperature
SCR: scrambled
SI: Signal Intensity
TNBC: Triple-negative breast cancer
WGA: Wheat Germ Agglutinin
