# Supplementary material for: Optimizing cisplatin delivery to triple-negative breast cancer through novel EGFR aptamer-conjugated polymeric nanovectors
Source: J Exp Clin Cancer Res. 2021 Jul 22;40:239. doi: 10.1186/s13046-021-02039-w (PMC8299618; doi:10.1186/s13046-021-02039-w)
Supplement: Supplementary file 1 — Additional file 1: [file 13046_2021_2039_MOESM1_ESM.pdf]

Supplementary Information

Purification and characterization of Cis-Pt@PNPs, Cis-Pt@PNPs-CL4 and Cis-Pt@PNPs-SCR

The Cis-Pt@PNPs, conjugated or not with CL4 and SCR, were washed and concentrated by using centrifugal filter devices (Amicon Ultra, Ultracell membrane with 100.000 NMWL, Millipore) to a final volume of 5 ml and finally filtered by using a syringe filters of nylon (Ø = 13 mm, 0.22 μm, Nazionale, Italy). Dynamic light scattering (DLS) analysis and ζ-potential values were obtained with a Zetasizer Nano-S (Malvern) instrument, working with a 532 nm laser beam at 25 °C, using standard cuvettes or DTS1070 Clear Disposable zeta cells, and the results expressed as average of three measurements. The DLS results reported that the Cis-Pt@PNPs were characterized by a diameter equal to 90.3 ± 0.3 nm, polydispersity index (PDI) of 0.254 and negative ζ-potential value of -21.4 mV. Cis-Pt@PNPs-CL4 and Cis-Pt@PNPs-SCR were characterized by a diameter equal to 91.1 ± 1.4 nm and 118.5 ± 2.0 nm respectively, PDI of 0.290 and negative ζ-potential value of -12.2 and 11.1 mV each.

The final concentrations of the suspensions were determined by gravimetric analysis by drying 100 μL of solution at 120 °C for 24 h then accurately weighting the residual dry matter amount. The results showed a final concentration of 7 mg/ml for all the three samples.

Quantitative determination of Cisplatin in Cis-Pt@PNPs, Cis-Pt@PNPs-CL4 and Cis-Pt@PNPs-SCR

In order to quantify the amount of Cis-Pt entrapped in the polymeric nanoparticles, microwave plasma atomic emission spectrometry has been carried out with an Agilent 4210 MP-AES equipped with nitrogen plasma gas (T up to 5000K). Pt standards solution were prepared diluting the Platinum Standard for AAS TraceCERT® (1000 mg/l Pt in hydrochloric acid) in aqua regia solution (8% v/v), to give the following final concentrations: 0.500; 1.00; 2.00; 5.00; 10.0; 50.0 mg/l. The standard solutions were freshly prepared before each analysis session and measured at λ 265.945 nm and 299.796 nm in order to create a calibration line. Cis-Pt@PNPs, Cis-Pt@PNPs-CL4 and Cis-Pt@PNPs-SCR were dissolved in aqua regia, then water is added until a dilution of 1:100 is reached and finally the samples are analysed.

The concentration of the entrapped cisplatin in Cis-Pt@PNPs resulted to be 460.78 μM (0.138 μg/μl), while in Cis-Pt@PNPs-CL4 was equal to 422.70 μM (0.127 μg/μl) and in Cis-Pt@PNPs-SCR was 455.18 μM (0.136 μg/μl).

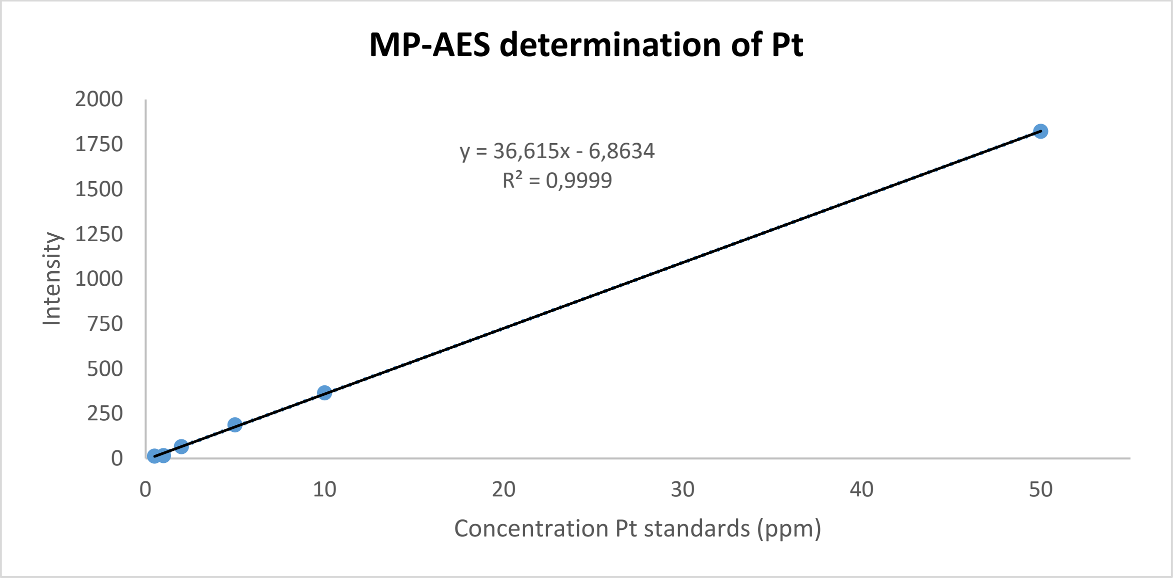

Figure S1. Quantification of entrapped Cisplatin through MP-AES determination of Pt. The calibration line has been obtained with the intensity measured for different molar concentrations of Pt (here is reported the λ=265.945 nm line).

Purification and characterization of BODIPY@PNPs, BODIPY@PNPs-CL4 and BODIPY@PNPs-SCR

The BODIPY containing PNPs, conjugated or not with CL4 and SCR, were washed and concentrated by using centrifugal filter devices (Amicon Ultra, Ultracell membrane with 100.000 NMWL, Millipore) to a final volume of 5 ml and finally filtered by using a syringe filters of nylon (Ø = 13 mm, 0.22 µm, Nazionale, Italy). Dynamic light scattering (DLS) analysis and ζ-potential values were obtained with a Zetasizer Nano-S (Malvern) instrument, working with a 532 nm laser beam at 25 °C, using standard cuvettes or DTS1070 Clear Disposable zeta cells, and the results expressed as average of three measurements. The DLS results reported that the BODIPY@PNPs were characterized by a diameter equal to 97.6 ± 0.3 nm, polydispersity index (PDI) of 0.142 and ζ-potential value of 0.08 mV. BODIPY@PNPs-CL4 and BODIPY@PNPs-SCR were characterized by a diameter equal to 131.9 ± 0.2 nm and 142.2 ± 1.5 nm respectively, PDI of 0.185 and negative ζ-potential value of -17.9 and -23.4 mV each.

The final concentrations of the suspensions were determined by gravimetric analysis by drying 100 µL of solution at 120 °C for 24 h then accurately weighting the residual dry matter amount. The results showed a final concentration of 8 mg/ml for BODIPY@PNPs and 5 mg/ml for both BODIPY@PNPs-CL4 and BODIPY@PNPs-SCR.

Quantitative determination of BODIPY®505-515 in BODIPY@PNPs, BODIPY@PNPs-CL4 and BODIPY@PNPs-SCR

The amount of BODIPY®505-515 entrapped in the polymeric nanoparticles was assessed by fluorescence quantitative determination using an Edinburgh FLSP920 spectrofluorometer equipped with a 450 W Xenon arc Lamp. A volume of 100 µl of each kind of sample was diluted 50 times in Dimethyl sulfoxide (DMSO) and put in an ultrasonic bath for 30 min. This process was aimed to destroy the pre-formed polymeric micelles due to the presence of organic solvent resulting in the BODIPY release. The obtained solutions have been further diluted 50 times in DMSO and the

spectrofluorometric determination has been performed using an excitation wavelength of 368 nm and an emission wavelength of 512 nm and BODIPY®505-515 standards solution at different concentrations (10^{-8} to 10^{-5} M) in DMSO (Figure S2).

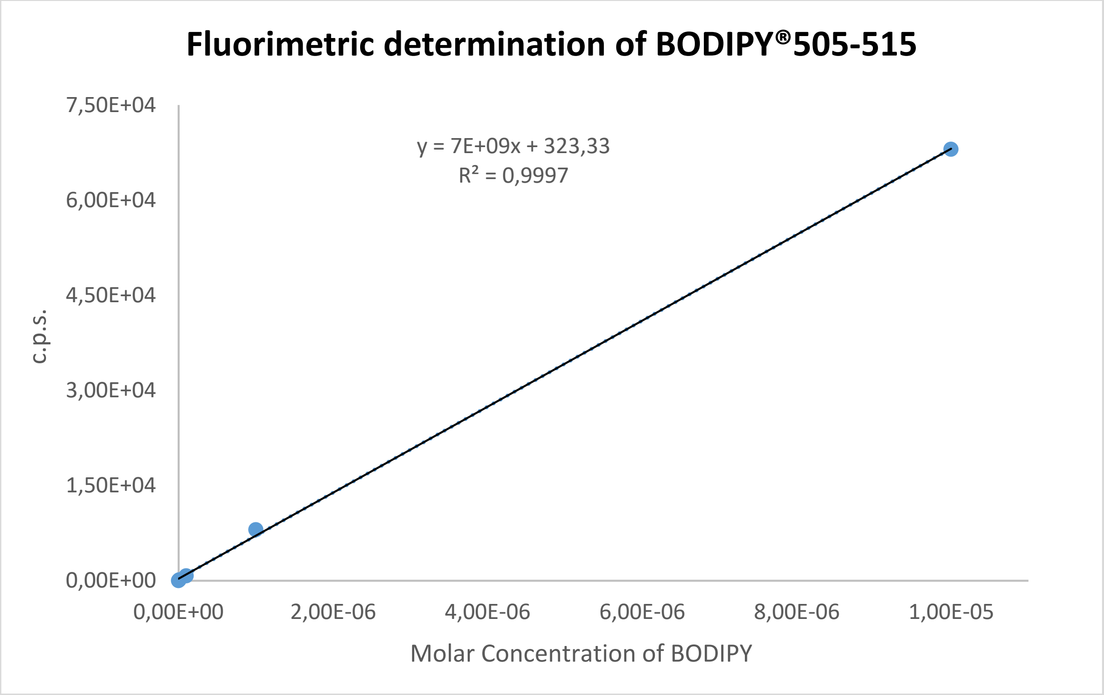

Figure S2. Fluorimetric determination of BODIPY®505-515. The calibration line has been obtained with the conts per second (c.p.s.) measured for different molar concentrations of BODIPY®505-515 in DMSO (λem = 512 nm).

The concentration of the entrapped BODIPY®505-515 in BODIPY@PNPs resulted to be 166 µM, while the concentration in BODIPY@PNPs-CL4 and BODIPY@PNPs-SCR resulted to be respectively 13.6 µM and 9.7 µM. The higher amount of dye in the first sample is due to the different centrifugal filter steps undertaken by the aptamers-conjugated samples.

Purification and characterization of Cy7@PNPs, Cy7@PNPs-CL4 and Cy7@PNPs-SCR

The Cy7 labelled PNPs, conjugated or not with CL4 and SCR, were washed and concentrated by using centrifugal filter devices (Amicon Ultra, Ultracell membrane with 100.000 NMWL, Millipore) to a final volume of 5 ml and finally filtered by using a syringe filters of nylon (Ø = 13 mm, 0.22 µm, Nazionale, Italy). Dynamic light scattering (DLS) analysis and ζ-potential values were obtained with a Zetasizer Nano-S (Malvern) instrument, working with a 532 nm laser beam at 25 °C, using standard cuvettes or DTS1070 Clear Disposable zeta cells, and the results expressed as average of three measurements. The DLS results reported that the Cy7@PNPs were characterized by a diameter equal to 90.5 ± 0.5 nm, polydispersity index (PDI) of 0.218 and ζ-potential value of -24.9 mV. Cy7@PNPs-CL4 and Cy7@PNPs-SCR were characterized by a diameter equal to 107.9 ± 0.3 nm and 104.2 ± 2.7 nm respectively, PDI of 0.350 and negative ζ-potential value of -20.6 mV.

The final concentrations of the suspensions were determined by gravimetric analysis by drying 100 µl of solution at 120°C for 24 h then accurately weighting the residual dry matter amount. The results showed a final concentration of 4 mg/ml for Cy7@PNPs and Cy7@PNPs-CL4 and 6 mg/ml for Cy7@PNPs-SCR.

Quantitative determination of Cy7@PNPs, Cy7@PNPs-CL4 and Cy7@PNPs-SCR

The amount of Cy7 conjugated to the carboxylic residues onto the polymeric nanoparticles' surface was assessed by absorbance quantification using an Agilent Cary 3500 Multicell UV-Vis spectrophotometer equipped with a Xenon flash lamp.

A volume of 100 µl of each kind of sample was diluted 20 times in H_2O and the spectrophotometric analysis was performed after a preliminary scan (λ range from 1100 to 400 nm): 745 nm was chosen as max absorption wavelength. Cy7 standards solution at different concentrations (0.5 to 5 µM) were prepared in H_2O (Figure S3).

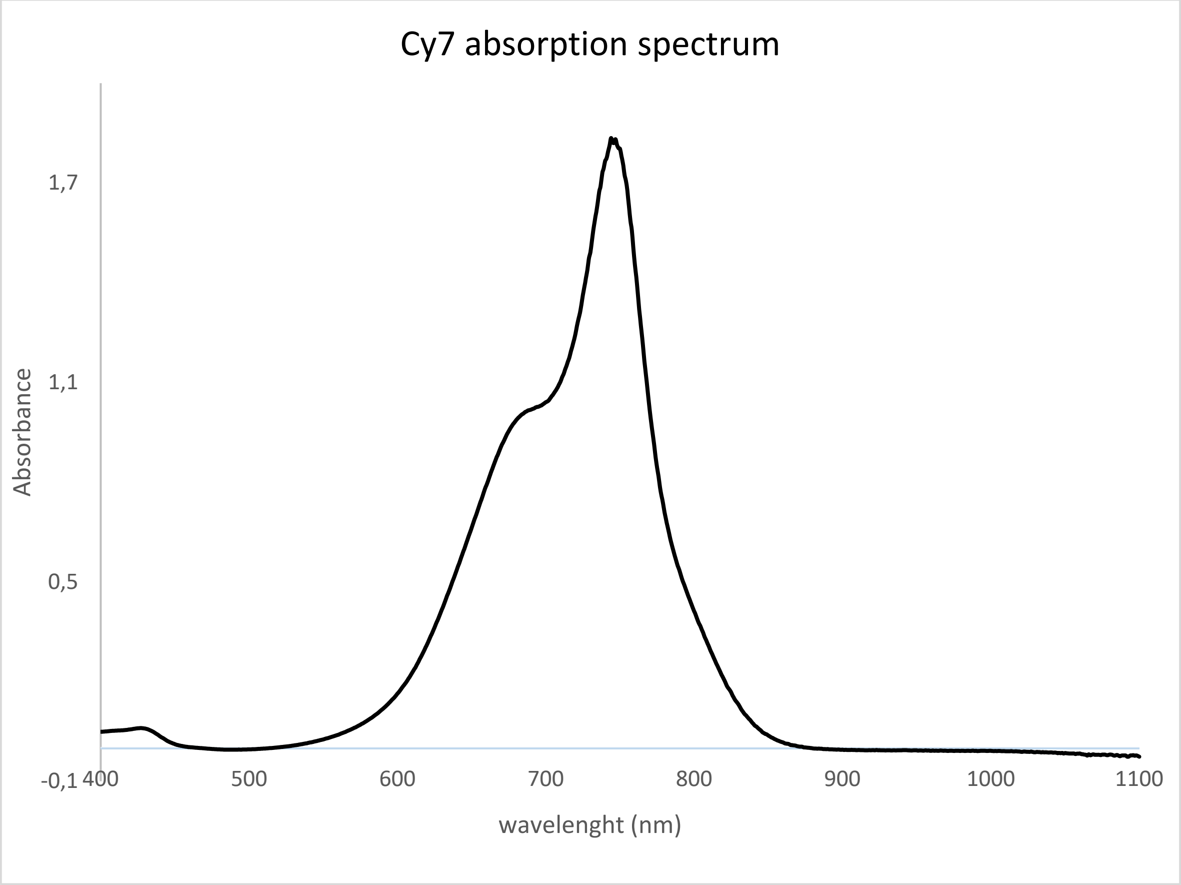

Figure S3. Determination of Cy7 by UV-Vis absorbance measurement (λabs max = 745 nm).

The concentration of the labelled Cy7 in Cy7@PNPs resulted to be 104.16 µM, while the concentration in Cy7@PNPs-CL4 and Cy7@PNPs-SCR resulted to be respectively 106.08 µM and 105.96 µM, indicating a > 90% of conjugation efficiency.

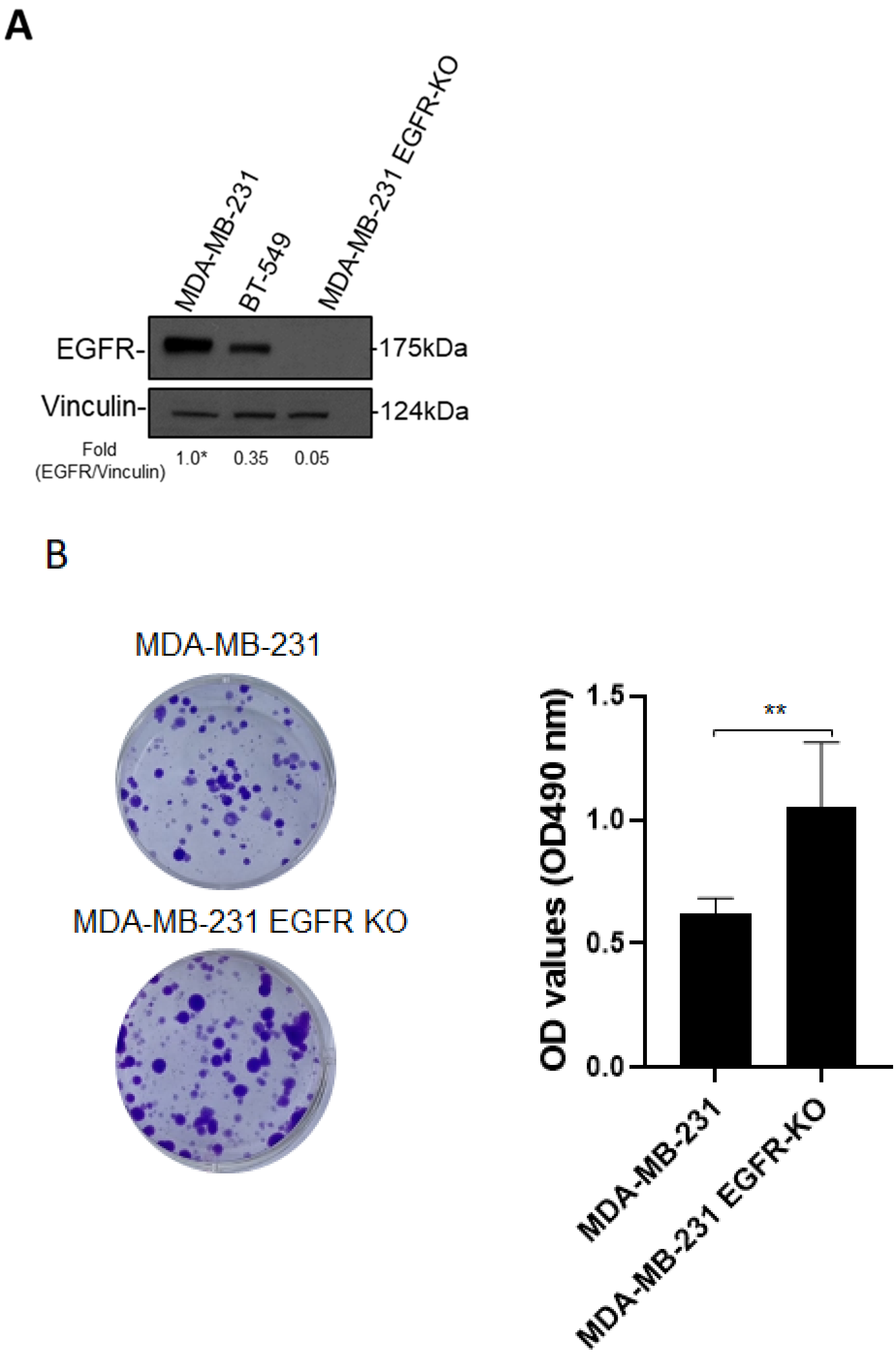

Figure S4. EGFR expression in TNBC cells. (**a**) Lysates from MDA-MB-231, BT-549 and MDA-MB-231 EGFR-KO cells were immunoblotted with anti-EGFR antibodies. Vinculin was used as an internal control. Values below the blot indicate signal levels, normalized to the respective anti-vinculin signal level, and reported as relative to MDA-MB-231 cells, arbitrarily set to 1 (labelled with asterisk). (**b**) A clonogenic assay was performed on MDA-MB-231 and MDA-MB-231 EGFR-KO cells for 3 weeks. Left panel shows representative images and right panel shows quantification. Error bars represent mean ± SD of triplicate experiments; ** $P < 0.01$ (unpaired *t-test*).

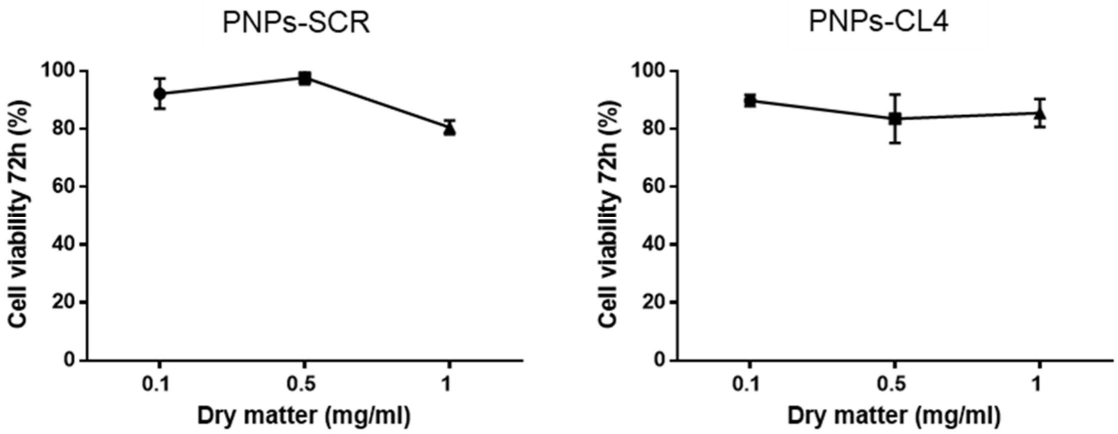

Figure S5. PNPs are not cytotoxic. MDA-MB-231 cells were mock-treated or treated for 72 h with the indicated amount of unloaded PNPs-SCR or PNPs-CL4. Cell viability was analyzed and expressed as percent of viable treated cells with respect to mock-treated cells. Each determination represents the average of three independent experiments and error bars represent SD.

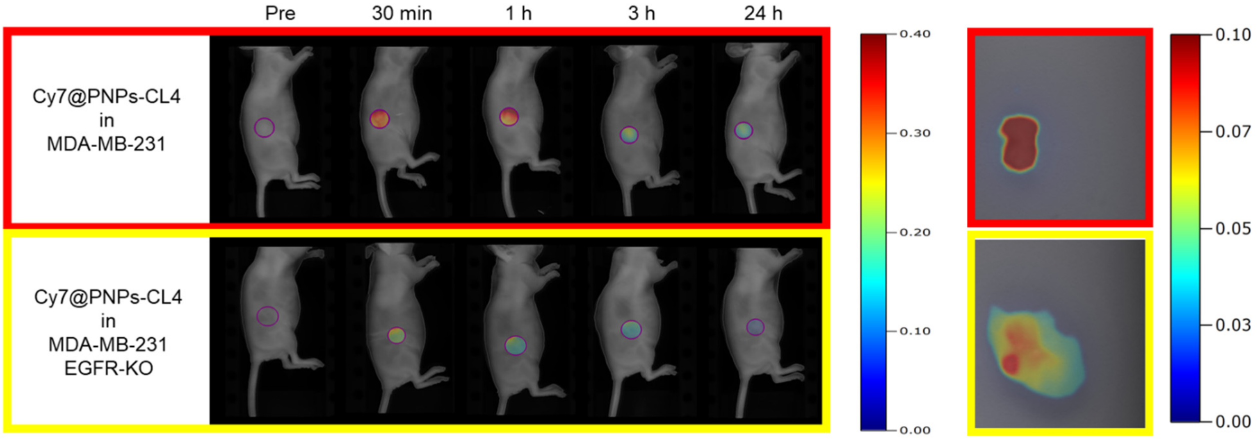

Figure S6. Selective imaging of EGFR-positive tumors by Cy7@PNPs-CL4. Mice bearing MDA-MB-231 (upper panels) or MDA-MB-231 EGFR-KO (lower panels) xenografts were *i.v.* injected with Cy7@PNPs (5 nmol Cy7) and analyzed by *in vivo* FRI at the indicated time points. Pre, before injection. On the right, *ex vivo* FRI of tumors excised from mice 24 h post-injection. The scale bar is in arbitrary units and is a colorimetric representation of the minimum and maximum signals; all the depicted images are reconstructed with the same scale. The normalized mean FRI Signal Intensity of EGFR-positive and EGFR-KO excised tumors was 4.3 and 2.1, respectively.
